# Loss of neuron network coherence induced by virus-infected astrocytes: a model study

**DOI:** 10.1038/s41598-023-33622-0

**Published:** 2023-04-19

**Authors:** Sergey V. Stasenko, Alexander E. Hramov, Victor B. Kazantsev

**Affiliations:** 1 Scientific—educational mathematical center “Mathematics of future technologies”, Lobachevsky University, Nizhniy Novgorod, Russia 603022; 2 Baltic Center for Artificial Intelligence and Neurotechnology, Immanuel Kant Baltic Federal University, Kaliningrad, Russia 236041; 3 Laboratory of neurobiomorphic technologies, Moscow Institute of Physics and Technology, Moscow, Russia 117303; 4 Neuroscience Research Institute, Samara State Medical University, Samara, Russia 443099

**Keywords:** Computational neuroscience, Glial biology

## Abstract

Coherent activations of brain neuron networks underlie many physiological functions associated with various behavioral states. These synchronous fluctuations in the electrical activity of the brain are also referred to as brain rhythms. At the cellular level, rhythmicity can be induced by various mechanisms of intrinsic oscillations in neurons or the network circulation of excitation between synaptically coupled neurons. One specific mechanism concerns the activity of brain astrocytes that accompany neurons and can coherently modulate synaptic contacts of neighboring neurons, synchronizing their activity. Recent studies have shown that coronavirus infection (Covid-19), which enters the central nervous system and infects astrocytes, can cause various metabolic disorders. Specifically, Covid-19 can depress the synthesis of astrocytic glutamate and gamma-aminobutyric acid. It is also known that in the post-Covid state, patients may suffer from symptoms of anxiety and impaired cognitive functions. We propose a mathematical model of a spiking neuron network accompanied by astrocytes capable of generating quasi-synchronous rhythmic bursting discharges. The model predicts that if the release of glutamate is depressed, normal burst rhythmicity will suffer dramatically. Interestingly, in some cases, the failure of network coherence may be intermittent, with intervals of normal rhythmicity, or the synchronization can disappear.

## Introduction

The synchronization of neuron network activity at the cellular and network levels gives rise to rhythmic voltage fluctuations traveling across brain regions, known as neuronal oscillations or brain waves^1,2^. Modulation of neural oscillations is provided by the dynamic interplay between neuronal connectivity patterns, cellular membrane properties, intrinsic circuitry, speed of axonal conduction, and synaptic delays^3–6^. The neural oscillations fluctuate between two main states, known as “up states” and “down states”^7^. The network coherence provided by the up state in spatially organized cortical neural ensembles plays a crucial role for several sensory and motor processes, as well as for cognitive flexibility (i.e., attention, memory), thereby playing a fundamental role in the brain’s basic functions^8–10^. Furthermore, different network dynamics (from slow to ultra-fast oscillations) can change according to the behavioral state, with some frequency bands being associated with sleep, while other frequencies predominate during arousal or conscious states^11–13^.

To study the mechanisms of synchronization of the neuron network activity at the cellular and network levels, a number of mathematical models have been proposed^14,15^. One approach is to consider the model of short-term synaptic plasticity as a possible synaptic mechanism for the formation of bursting activity^16–19^.

Besides purely neuronal mechanisms, many recent studies revealed the essential contributions made by astrocytes to many physiological brain functions,including synaptogenesis^20^, metabolic coupling^21^, nitrosative regulation of synaptic release^22–24^, synaptic transmission^25^, network oscillations^26^, and plasticity^27,28^. Astrocytes can play a significant role in the processing of synaptic information through impact on pre- and post-synaptic neurons. This fact leads to the concept of a tripartite synapse^29,30^. A part of the neurotransmitter released from the presynaptic terminals (i.e., glutamate) can diffuse out of the synaptic cleft and bind to metabotropic glutamate receptors (mGluRs) on the astrocytic processes that are located near the neuronal synaptic compartments. The neurotransmitter activates G-protein mediated signaling cascades that result in phospholipase C (PLC) activation and insitol-1,4,5-trisphosphaste (IP3) production. The IP3 binds to IP3-receptors in the intracellular stores and triggers $Ca^{2+}$ release into the cytoplasm. Such an increase in intracellular $Ca^{2+}$ can trigger the release of gliotransmitters^31^ [e.g., glutamate, adenosine triphosphate (ATP), D-serine, and GABA] into the extracellular space. A gliotransmitter can affect both the pre- and post-synaptic parts of the neuron. By binding to presynaptic receptors, it can either potentiate or depress presynaptic release probability. One of the key pathways in the tripartite synapse is mediated by glutamate released by the astrocyte^32–34^. Such glutamate can potentially target presynaptic NMDA receptors, which increase the release probability^35^, or presynaptic mGluRs, which decrease it^36^. Presynaptic kainate receptors exhibit a more complex modulation of synaptic transmission through both metabotropic and ionotropic effects^37,38^. Based on experimental data, many computational models have been proposed taking into account neuron to astrocyte interactions to describe the interneuronal communication^39–46^. Many experimental studies have shown that astrocytes can coordinate the neuronal network activations^45,47–49^. Because astrocyte is affected by a large number of synapses, the gliotransmission should also contribute to the effect of neuronal synchronization^50–54^. Particularly, it was demonstrated in a hippocampal network that calcium elevations in astrocytes and subsequent glutamate release led to the synchronous excitation of clusters of pyramidal neurons^55,56^.

Coronavirus SARS-CoV-2 has become a global challenge of the modern world, stimulating intensive research in many related areas of science. Along with the development of vaccines, a fundamentally important global task is to investigate Covid-19 effects on different systems of human organisms. Recent studies have shown that coronavirus infection, entering the central nervous system and infecting astrocytes, causes various metabolic disorders^57,58^, one of which is a decrease in the synthesis of astrocytic glutamate and gamma-aminobutyric acid (GABA)^57^. It is also known that in the postcovid state, patients may suffer from symptoms of anxiety and impaired cognitive functions^57,58^. In this paper, we propose a mathematical model of impact infected astrocyte on the ability to synchronize neural networks and produce brain rhythms. We show that depending on the degree of disturbance in the synthesis of gliotransmitters neuronal network synchronization can be partially or completely suppressed.

## Methods

### Classical model of single neuron

To describe the dynamics of a single neuron, we use the Izhikevich model^59^, which represents a compromise between computational complexity and biophysical plausibility. Despite its computational simplicity, this model can replicate a large number of phenomena that occur in real neurons. The Izhikevich model is formulated as a system of differential Eq. (1):

1 $$\begin{aligned} {\left\{ \begin{array}{ll} C\frac{dV_{i}}{dt} = k(V_{i} - V_{r})(V_{i} - V_{t}) - U_{i} + I_{ext_i} + I_{syn_i} ,\\ \frac{dU_{i}}{dt} = a(b(V_{i} - V_{r}) - U_{i}). \end{array}\right. } \end{aligned}$$

If $$V_{i} \ge V_{peak}$$, then

2 $$\begin{aligned} {\left\{ \begin{array}{ll} V_{i} = c, \\ U_{i} = U_{i} + d, \end{array}\right. } \end{aligned}$$

where *i* (*i*=1,...,*N*) corresponds to a neuronal index, $a, b, c, d, k, C, V_t and V_r$ are the different parameters of the neuron. $V_{i}$ is the potential difference between the inside and outside of the membrane, and $U_{i}$ is a “recovery variable” describing the process of activation and deactivation of potassium and sodium membrane channels, respectively. As a result, we have negative feedback that affects the dynamics of the potential $V_{i}$ on the cell membrane. The resting potential value in the model lies in the range from -70 to -60 mV. Its value is determined by the parameter *b*, which describes the sensitivity of the recovery variable to subthreshold potential fluctuations in the neuronal cell membrane. The parameter *a* sets the characteristic time scale of the change in the recovery variable *u*. The $V_{peak}$ value limits the spike amplitude. Parameters *c* and *d* specify the values of $V_{i}$ and $U_{i}$ after spike generation. $I_{ext_i}$ is the externally applied current. The neuron model is in an excitable mode and demonstrates the generation of spikes in response to an applied current. $I_{syn_i}$ represents the total synaptic current from all neurons with which this neuron is connected. The total synaptic current $I_{syn}$ received by neuron *i* from *M* presynaptic neurons was calculated as follows:

3 $$\begin{aligned} I_{syn_i} = \sum _{j=1}^{M} w_{i,j} y_{i,j}, \end{aligned}$$

where $w_{i,j}$ denotes the weights for glutamatergic and GABAergic synapses between neurons. For excitatory and inhibitory contacts, the weights have positive and negative signs, respectively. Variables $y_{i,j}$ denote the output signal (synaptic neurotransmitter) from the *j*-th neuron to the *i*-th neuron involved in the production of $I_{syn_i}$, *M* is the number of non-zero contacts. Note that the total number of synaptic connections is $N^{2} \times p$, where *N* is the number of neurons, *p* is the probability of communication between two random neurons , which is set to 0.1 (corresponding to $10 \%$ of connections). Each synaptic weight was set randomly for all connections in the range from 20 to 60. If a spike is generated on the presynaptic neuron, a jump in the synaptic current occurs on the postsynaptic neuron, which then decays exponentially. As a result, synaptic neurotransmitter concentration, $y_{i,j}$, was calculated as follows:

4 $$\begin{aligned} y_{i,j}(t) = \left\{ \begin{array}{lcl} y_{i,j}(t_i)exp(-t/\tau _y) &{} \text{ if }, &{} t_s<t<t_{s+1}, \\ y_{i,j}(t_s-0)+1 &{} \text{ if }, &{} t=t_s, \end{array} \right. \end{aligned}$$

where $t_s$ denotes the time moments of consequent presynaptic spikes, $\tau _y$ is a relaxation time constant.

Each spike in the neuron model induces the release of neurotransmitter. To describe the neuron to astrocyte cross-talk, here we only focus on the excitatory neurons releasing glutamate. Following earlier experimental and modeling studies, we assumed that the glutamate-mediate exchange was the key mechanism to induce coherent neuronal excitations^55,56^. The role of GABAergic neurons in our network is to support the excitation and inhibition balance avoiding hyperexcitation states.

For simplicity, we take a phenomenological model of released glutamate dynamics. In the mean-field approximation average concentration of extyrasynaptic glutamate concentration for each excitatory synapses, $X_{e}$, was described by this equations:

5 $$\begin{aligned} X_{e}(t) = \left\{ \begin{array}{lcl} X_{e}(t_s)exp(-t/\tau _X), &{} \text{ if } &{} t_s<t<t_{s+1}, \\ X_{e}(t_s-0)+1, &{} \text{ if } &{} t=t_s, \end{array} \right. \end{aligned}$$

where $e=1,2,3,...$ is the index of excitatory presynaptic neurons, $s=1,2,3,\ldots$ is the index of the presynaptic spikes, $\tau _{X}$ is the time relaxation. After a spike is generated on the presynaptic neuron, the neurotransmitter is released, and the concentration of the extrasynaptic neurotransmitter increases due to diffusion processes, but it decreases over time with its characteristic time constant,$\tau _{X}$. So that, the difference in mathematical descriptions of synaptic (4) and extrasynaptic (5) is accounted for by the different time constants $\tau _y$ and $\tau _X$, respectively.

### Dynamics of astrocytic signal

**Figure 1:**
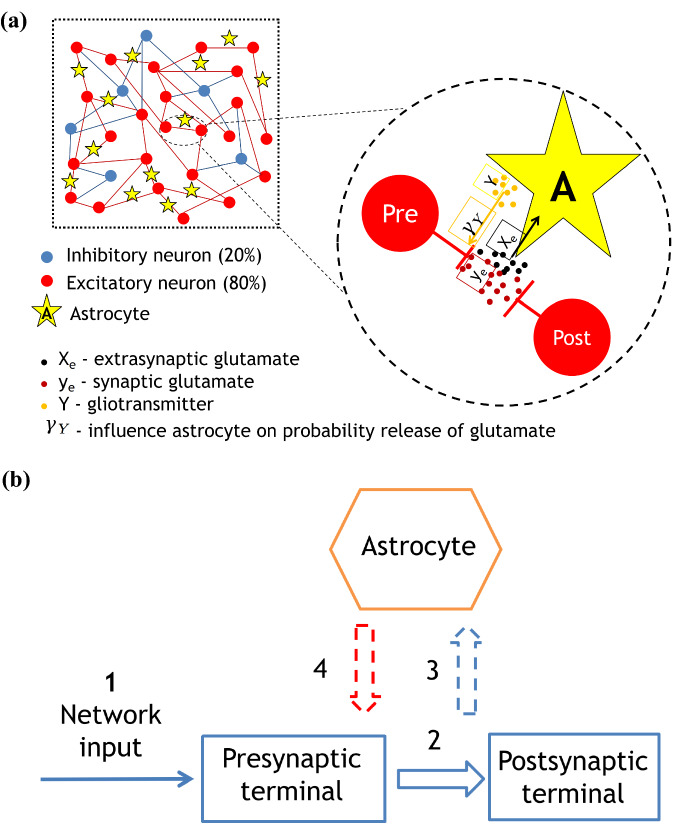
(**a**) - Schematic view of the network and a schematic representation of astrocytic modulation of synaptic current, (**b**) - Diagram of the sequence of influences and interactions in a tripartite synapse: 1 - input from the neural network to the presynaptic terminal, 2 - release of the neurotransmitter, 3 - diffusion of the neurotransmitter and binding to receptors on the astrocyte membrane, 4 - release of the gliotransmitter from the astrocyte and its effect on the presynaptic terminal through a change in the probability of neurotransmitter release.

Part of the extrasynaptic glutamate can bind to metabotropic glutamate receptors on the astrocyte processes. Subsequently, after a cascade of molecular transformations mediated by an elevation of intracellular calcium, the astrocyte release gliotransmitter back to the extracellular space. For our purpose, in the framework of qualitative mean-field description, we have omitted molecular details of these transformations, and instead defined only the input-output functional relation between the neurotransmitter and gliotransmitter concentrations, as follows^43,46,51,54^:

6 $$\begin{aligned} \frac{dY_{e}}{dt} = - \alpha _{Y}Y_{e} + \frac{\beta _{Y}}{1+exp(-X_{e} + X_{thr})} \end{aligned}$$

where $e=1,2,3,\ldots$ is the index of excitatory neuron, $Y_e$ is the gliotransmitter concentration in the neighborhood of the corresponding excitatory synapse, $\alpha _{Y}$ is the clearance rate and $\beta _{Y}$ is the release rate. So that, the second term in Eq. (2) describes the gliotransmitter production when the mean-field concentration of gliotransmitter exceeds some threshold, $X_{thr}$. Figure 1 illustrates the network construction and neuron-to-astrocyte crosstalk for excitatory glutamatergic synapses.

We also considered the effect of the astrocyte infection. Recent experimental studies demonstrated that COVID-19 infection results in a decrease in astrocytic glutamate and GABA synthesis^57^. For our purposes, this means that the amount of released gliotransmitter locally decreases with the overall level of infection. In the mean-field approach, this can be modeled by scaling the release rate as follows:

7 $$\begin{aligned} \beta _{Y} =\beta ^0_{Y} (1 - \gamma _{virus}), \end{aligned}$$

where $\beta ^0_{Y}$ represents the release rate for non-infected astrocyte and $0< \gamma _{virus}<1$ is the scaling coefficient. Phenomenologically, quantity $\gamma _{virus}$ can be treated as the infection probability for local astrocyte. In a “spatial” treatment $\gamma _{virus}$ describes the fraction of infected astrocytes in the whole ensemble and can be associated with the level of viral load. In the limit cases of well-functioning cell $\gamma _{virus}=0$, it takes unity value and the production rate is accounted by $\beta _Y$, while for totally infected cells, $\gamma _{virus}=0$, it takes zero value and no release happens at all.

It should be noted that the depression of gliotransmitter release might not be specific to Covid action only. The release process is regulated by a complex cell molecular machinery that can not be fully accounted for in the framework of phenomenological models. However, such Covid-associated local astrocyte dysfunction, accounted in the mean-field model by the release scaling, will result in global changes in neural circuit dynamics at the network level.

### Astrocytic modulation of neural activity

It follows from experimental evidence that astrocytes can influence the probability of neurotransmitter release^34,60,61^, which in turn results in modulation of synaptic currents. We take this into account in the following form for glutamatergic synapses:

8 $$\begin{aligned} I_{syn_i} = \sum _{j=1}^{M} w_{i,j} y_{i,j} (1+\gamma _{Y} \cdot Y_e) \end{aligned}$$

where $I_{syn_i}$ is the sum of all synaptic currents of the postsynaptic neuron, $w_{i,j}$ is the weight for glutamatergic synapses between neurons, $\gamma _{Y}$ is the coefficient of astrocyte influence on synaptic connections.

### Spiking neural network

Schematic representation of the network with astrocytic modulation of the probability release of neurotransmitter is presented in Fig. 1a. After the generation of an action potential on the presynaptic neuron, the neurotransmitter is released from the presynaptic terminal. A part of it can diffuse out of the cleft, where it can bind to specific astrocyte receptors^62^. The activation of the astrocyte results in the generation of calcium transients in the form of short-term increases in the intracellular concentration of calcium. In turn, the calcium elevations lead to gliotransmitter (particularly glutamate) release. The released gliotransmitter, upon reaching the presynaptic terminal, leads to a change in the probability of neurotransmitter release, potentiating the synaptic current. Fig. 1b shows a diagram of the sequence of influences and interactions in a tripartite synapse: 1 - input from the neural network to the presynaptic terminal, 2 - release of the neurotransmitter, 3 - diffusion of the neurotransmitter and binding to receptors on the astrocyte membrane, 4 - release of the gliotransmitter from the astrocyte and its effect on the presynaptic terminal through a change in the probability of neurotransmitter release. This, in turn, leads to the formation of burst activity.

**Figure 2:**
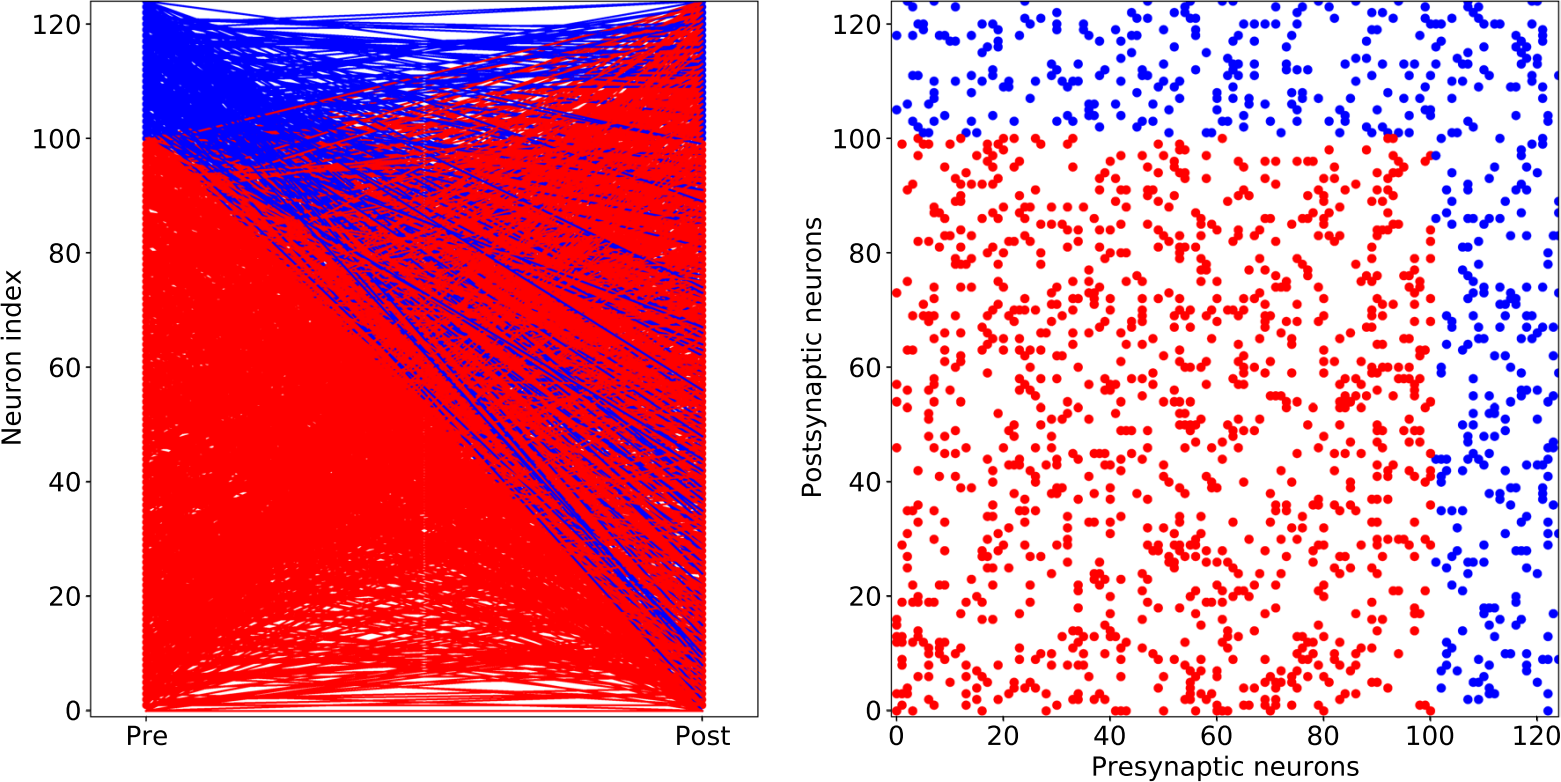
Scheme of synapse connections in neural networks. The left panel shows the connections between pre and postsynaptic neurons. Neurons on the vertical axis are ordered with excitatory ones, $N_{ex}$, coming first followed by the inhibitory ones, $N_{inh}$. The synaptic connections are illustrated by lines from the left (“Pre”) to the right (“Post”) in the figure. Red lines denote the excitatory connections, and the blue lines correspond to the inhibitory ones. The figure on the right shows connectivity matrix, $w_{i,j}$, with coordinates according to the numbers of pre- and postsynaptic neurons. Each dot in the field denotes the presence of nonzero synaptic connections.

The architechture of synaptic connections in our model neywork is illustrated in Fig. 2. The left panel shows the connections between pre- and postsynaptic neurons. Neurons on the vertical axis are ordered with excitatory ones, $N_{ex}$, coming first followed by the inhibitory ones, $N_{inh}$. The synaptic connections are illustrated by lines from the left (“Pre”) to the right (“Post”) in the figure. Red lines denote the excitatory connections, and the blue lines correspond to the inhibitory ones. The figure on the right shows connectivity matrix, $w_{i,j}$, with coordinates according to the numbers of pre- and postsynaptic neurons. Each dot in the field denotes the presence of nonzero synaptic connections.

In our simulations, we used $N=125$ spiking cortical neurons with 1562 synaptic connections in real time (resolution 1 ms). Motivated by the anatomy of a mammalian cortex, we choose the ratio of excitatory to inhibitory neurons to be 4 to 1. Thus, we took $N_{ex}=100$ and $N_{inh}=25$, respectively. Besides the synaptic input, each neuron receives a noisy thalamic input ($I_{ext_i}$). The noisy thalamic input is set randomly for all neurons in the range from 0 to 50. Since the model uses a mean-field approach to describe changes in the main neuroactive substances (neurotransmitter and gliotransmitter), we do not separate the effect of a single astrocyte on a group of neurons or a group of neurons on a single astrocyte, but we introduce into the description of each synaptic contact its own dynamics for the neuro and gliotransmitter.

For neuronal network simulation, we took parameters typically used in simulations of Izhikevich’s neurons in a spiking mode. The astrocyte was modeled phenomenologically, and the parameters were tuned to generate population bursts as a normal state of the network without infection.

We simulated the model by performing numerical integration of Eqs. (1) to (7) using the Euler method with a time step of 0.5 ms. Such a procedure has been shown to be appropriate for integrating large systems of the Izhikevich’s neurons^59,63^. The software used for simulation was written in the object-oriented programming language C++.

### Network signaling characteristics

**Figure 3:**
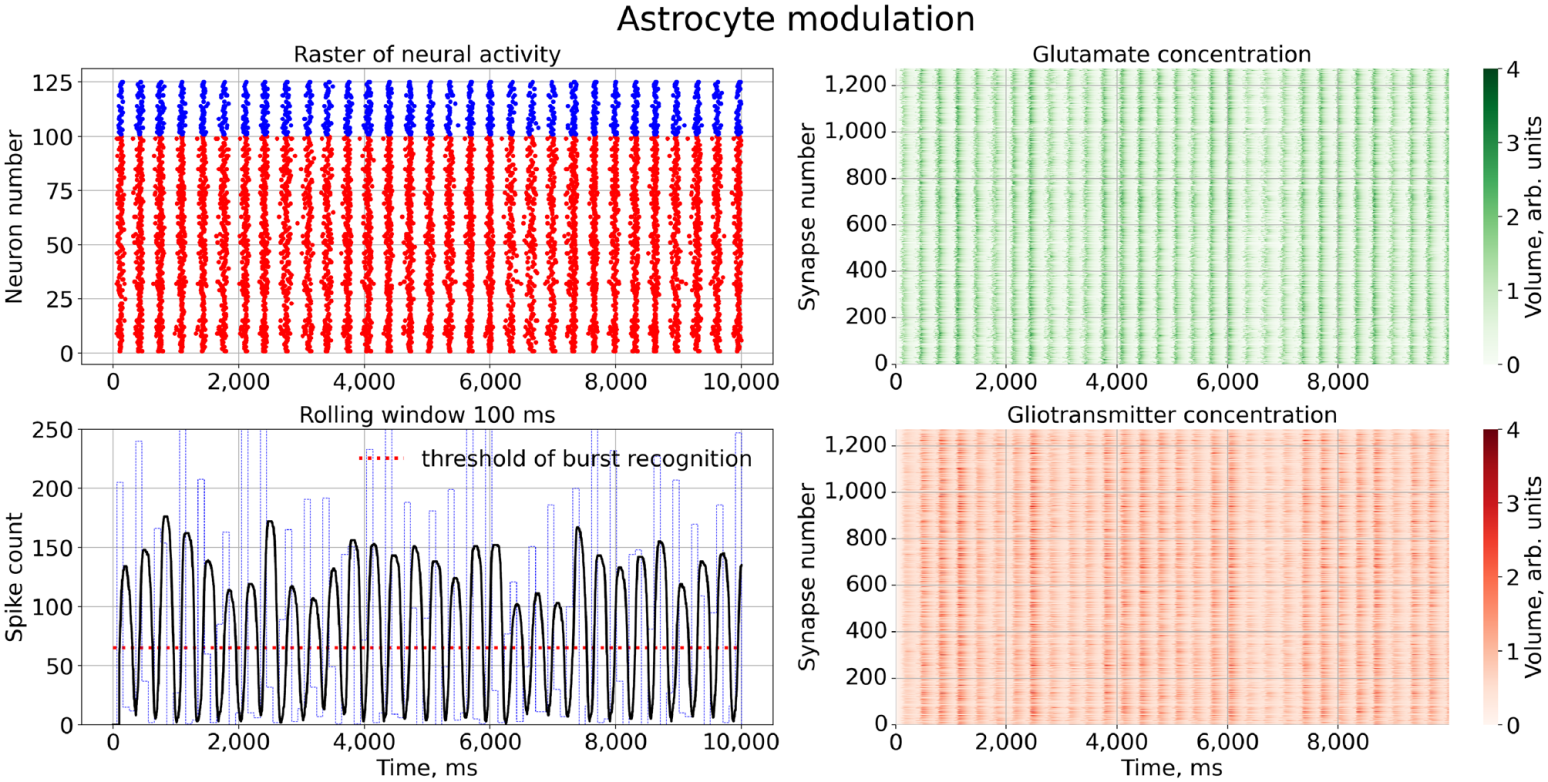
Network firing under normal conditions. Left upper panel: A raster plot of neural activity, with red dots indicating spikes by excitatory (glutamatergic) piramidal neurons and blue dots indicating spikes by inhibitory (GABAergic) interneurons. Right panels: Changes in the extracellular concentrations of synaptic glutamate diffused from the cleft (green color) and the glutamate released by astrocyte (red color) for all tripartite synapse. Left lower panel: the count of spikes over a sliding time window of 100 ms is shown for the entire simulation time of the model. We set the burst generation threshold at 65 spikes, marked by a red dashed line. The parameter values used are: for neuron - $a = 0.02, b = 0.5, c = -40, d = 100, k = 0.5, C = 50, V_{r} = -60, V_{peak} = 35, V_{0} = -60, U_{0} = 50$; other - $\tau _y = 4, \tau _X = 100, \alpha _{Y} = 80, \beta _{Y} = 1, X_{thr} = 5.6, \gamma _{Y} = 0.72$.

We trained the network model to generate the so-called population burst dynamics, which represent time intervals of quasi-synchronous spiking alternating with irregular spiking^64–66^. A typical example is shown in Fig. 3. Generally, such signalling may emerged from modulations of the weights of excitatory and inhibitory neuronal populations. For example, this modulation can be associated with short-term plasticity in local synapses^16–19^. Briefly, such short-term synaptic changes are frequency dependent, and after high-frequency firing, the neurotransmitter release is suppressed. Here, we consider a different situation where local synapses were supplied with astrocytes regulating the neurotransmitter release via calcium activation and consequent gliotransmitter release. Note, that population bursting is quite different from single cell bursting generated by other neuronal models, such as the Hindmarch-Rose neuron. Without coupling, each neuron works in its spiking mode.

To characterize the bursting dynamics, we used the following calculation algorithm:

- The number of neural network spikes, $N_{spikes}$, was counted for all neurons, *N*, at a time, $t = 1$ ms, $N_{spikes} = \sum _{i=1}^{N} S(V_i)$, where $$S(V_i)= {\left\{ \begin{array}{ll} 1,&{} \text {if } V_i > V_{peak}\\ 0, &{} \text {otherwise} \end{array}\right. }$$.
- The number of spikes in the sliding time window,$T_W = 100$ ms, was counted $W_{spikes} = \sum _{t=1}^{T_W} N_{spikes}$.
- The sliding time window moves to $t + 1$ and the number of spikes is recalculated.
- According to the threshold of bursting generation (in Fig. 3 marked with a red line), determined by the burst mode of neural activity, the frequency of bursts is calculated.

Next, we calculated the graph of the dependence of the burst frequency on a model parameter. Each point of the graph was obtained by averaging 1000 simulation experiments. To estimate the regression curve, we used the following equation:

9 $$\begin{aligned} y = A \exp \left( -\left( \frac{x - B}{C}\right) ^2 + D\right) , \end{aligned}$$

where *A*, *B*, *C*, *D* are parameters of the regression equation, *x* is the data vector obtained for the numerical experiment.

## Results

First, let us consider how the astrocytes induced the appearance of quasi-synchronous bursting dynamics. If no astrocytic feedback is activated, e.g., $\gamma _{Y}=0$, the network showed asynchronous spontaneous firing due to the uncorrelated noisy component of applied current, $I_{ext_i}$, which stimulates all neurons (not shown in the figures). When the feedback is activated, $\gamma _{Y}>0$, the model starts to generate population burst discharges, as illustrated in Fig. 3.

Similar to previous modeling studies^45,47–49^ the astrocytes started to coordinate neuronal activity, inducing a certain level of coherence in the network firing. On the one hand, each astrocyte is activated by integrating neuronal activity in its neighbouring space. On the other hand, when astrocyte is activated, it facilitates the synchronous activation of accompanying neurons within a certain area. As a result, neurons generated quasi-synchronous high-frequency burst discharges (Fig. 3). These discharges are synchronized with peaks of extracellular glutamate concentration associated with the astrocytes activations. It should be noted that population burst dynamics is typical for living networks formed in dissociated cortical (or hippocampal) neuronal culture models in vitro^64,67,68^. In such biological models, *normal* bursting indicates normal activity. In different pathological conditions (hypoxic-ischemic injury, alpha or theta coma or electrocerebral inactivity^69^) bursting fails, which indicates the decrease of functional coherence in the network firing.

**Figure 4:**
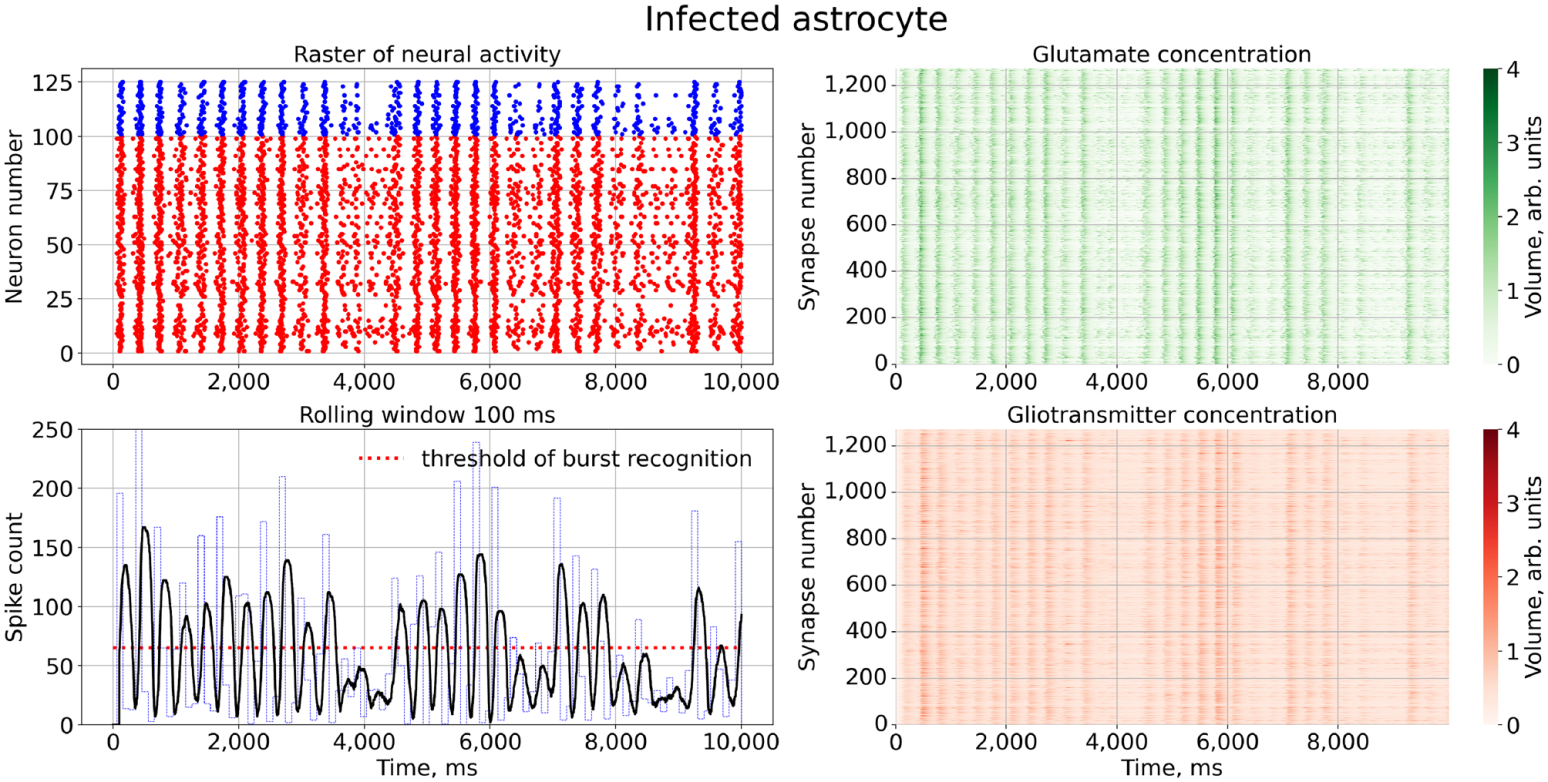
Raster chart of neural activity and dependences of gliotransmitter and neurotransmitter concentration from time with infected astrocyte feedbacks for $\gamma _{virus}=0.10$.

Next, we activated the virus pathological action in the model by increasing $\gamma _{virus}>0$. Figure 4 illustrates how network activity changes in this case. The raster plot shows that normal bursting were interrupted by the intervals of asynchronous uncorrelated firing. Corresponding graphs of glutamate concentration in the right panels indicate that in these intervals the astrocytes were partly (lower peaks) or completely (no peaks) inhibited. After this intervals bursts were spontaneously recovered to normal sequences. So, the result of the astrocyte infection at network level provokes the failure of normal synchronization at the network level, while each neuron in the network works fine, and each synaptic connections stay well-functioning. Note that, for low values of $\gamma _{virus}$ associated with “light” infection cases, the intervals of uncorrelated firing are quite shot, indicating a kind of intermittent behavior between long-lasting normal synchronous (e.g. “laminar”) stages and rather shot pathological asynchronous (e.g. “turbulent”) breaks.

**Figure 5:**
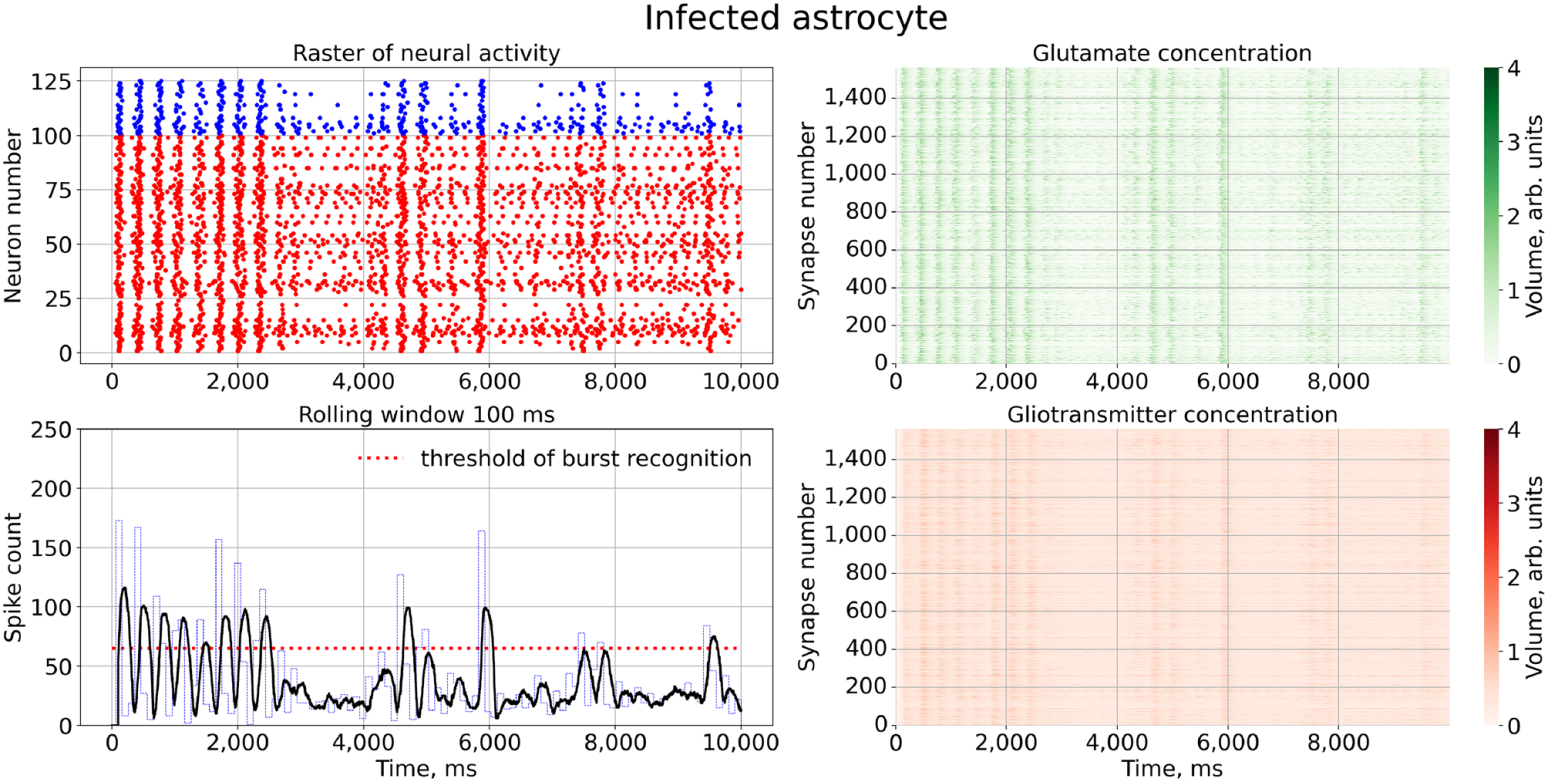
Raster chart of neural activity and dependences of gliotransmitter and neurotransmitter concentration from time with infected astrocyte feedbacks for $\gamma _{virus}=0.2$.

The next prediction of the model concerns the gradual character of the infection’s influence. This means that higher concentration of the virus in the organism will result in a stronger pathological response. In terms of our model, an increase in $\gamma _{virus}$ leads to an increase in the intervals of “pathological” firing (Fig. 5). One can note that the number of normal bursts within the same sample window significantly decrease. In terms of the concentrations of neuro- and gliotransmitter (right panels of Fig. 5), we also noticed a decrease in functionality not only of all astrocytes but also neurons. Some of them become depressed due to the lack of a sufficient amount glutamate to support normal excitatory transmission. Therefore, the higher virus concentration is exposed, then more astrocytes are infected and, hence, more “explicit” pathological synchrony breaks appear at the level of network firing.

**Figure 6:**
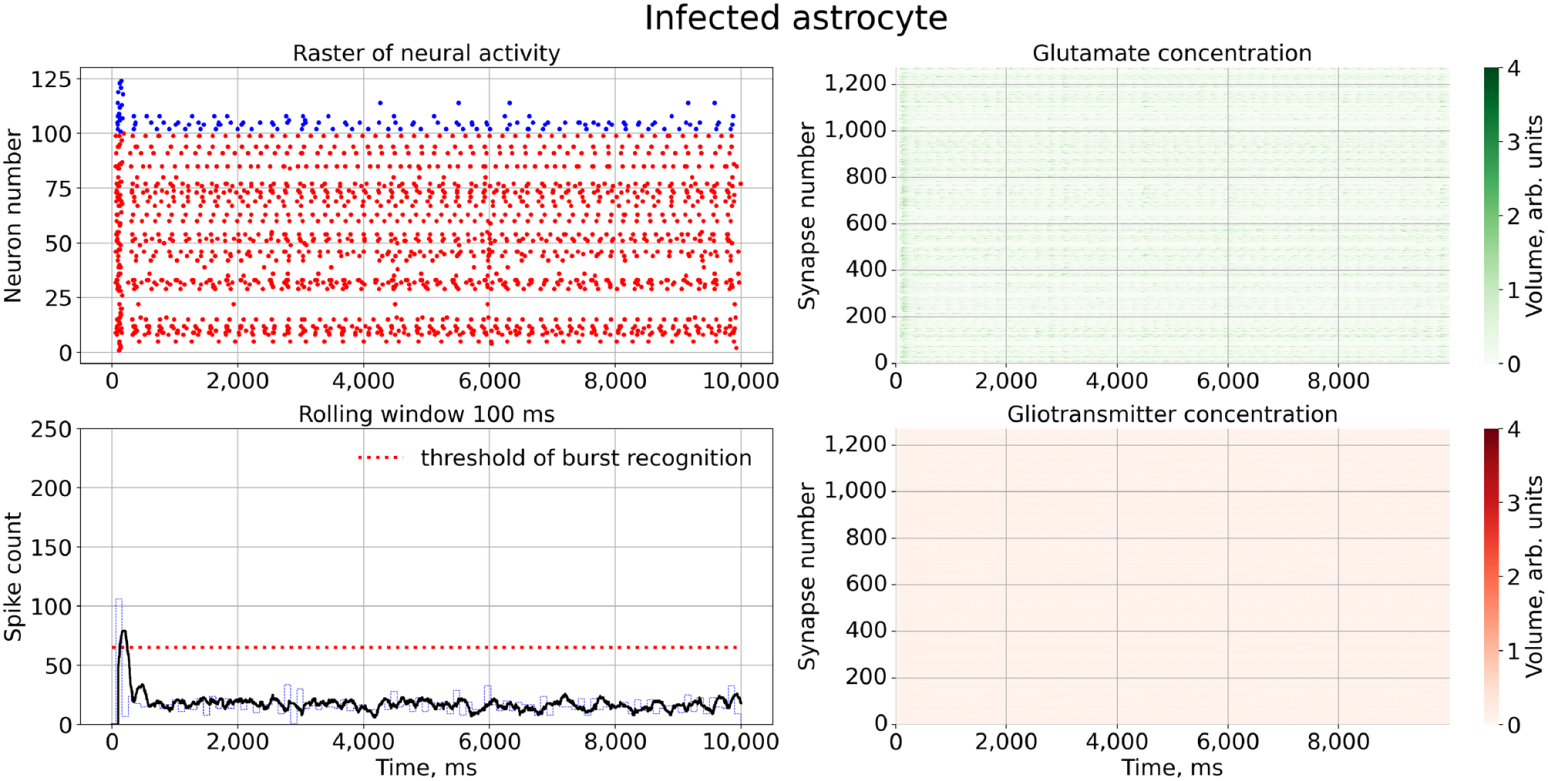
Raster chart of neural activity and dependences of gliotransmitter and neurotransmitter concentration from time with infected astrocyte feedbacks for $\gamma _{virus}=0.8$.

**Figure 7:**
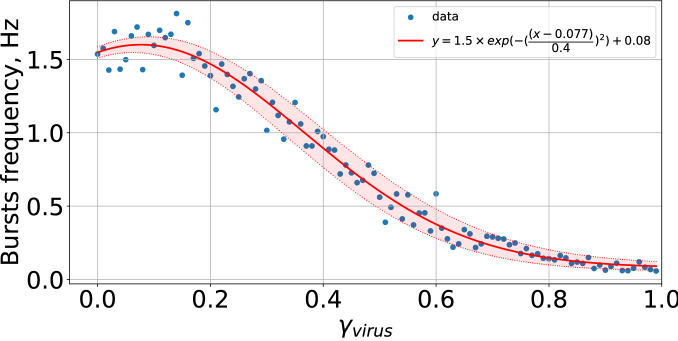
Dependence of bursts frequency from $\gamma _{virus}$.

As one may expect now, further increase in $\gamma _{virus}$ completely inhibit the synchronization. This is illustrated in Fig. 6. Correspondingly, all astrocytes fail to release any glutamate. However, note that overall network firing still persists, sustained by activations of excitatory neurons with relatively strong glutamatergic synapses. To quantify the gradual character of network dysfunction due to the infection we calculated a quantity reflecting the average burst frequency versus $\gamma _{virus}$ (Fig. 7). The graph represents monotonically descreading function, vanishing at $\gamma _{virus} \rightarrow 1$.

## Discussion

At the cognitive level, normal brain function is associated with functional networks that involve long-range correlations between different groups of neurons, each responsible for a particular function^70,71^. Failure of these correlations may indicate the appearance of cognitive dysfunctions^72^.

At the cellular level, functional synchronization is achieved through coherent firing patterns of spiking neuronal circuits. Our mathematical model, following in vitro biological models of neuronal cultures where the appearance of population bursts provides functional synchronization, predicts that infected (in particular, infected by Covid-19 virus) astrocytes might be responsible for the failure of functional synchronization and consequent cognitive dysfunctions.

It was also found from our model study that the change in the period of the “laminar phase”, i.e. the duration of regular bursts, and the duration of asynchronous states (say, the “turbulent phase”) is controlled by the $\gamma _{virus}$ parameter (taking into account the level of astrocyte dysfunction), as shown in Fig. 7. In this context, the duration of the “turbulent” phase (e.g. without bursting) can be associated with temporal cognitive state of mental dysfunction. Increasing the virus load (accounted in the model by gamma) induces longer periods of dysfunctions. Recovering the gliotransmitter release immediately recovers normal bursting rhythmicity.

At present, cognitive dysfunctions are reported as one of the most dangerous consequences of Covid-19 infection in post-covid states (patients may suffer from symptoms of anxiety and impaired cognitive functions^57,58^). Patients’ EEG scans show a range of abnormalities in brain activity, including some rhythmic patterns and epileptic-like spikes in activity^73^. Therefore, we hope that our developed model and the results obtained can clarify the processes occurring in the body after Covid-19 infection.

## Conclusion

We constructed spiking neuron network that communicates with astrocytes. For a certain choice of model parameters, the network demonstrated signals in the form of robust quasi-synchronous population bursts, which we treated as the basic or normal state of the network. The model takes into account astrocyte activation depending on the integrative level of neuronal firing and the astrocyte-to-neuron feedback that is based on the released gliotransmitter (glutamate), which facilitates group firing of neurons within the astrocyte territory. Following experimental facts, we assumed that infected astrocytes (in particular, infected by Covid-19) suffer from decreased gliotransmitter release. We accounted this fact by incorporating feedback that depends on the viral load.

Next, the model predicted qualitative changes in neuronal network firing depending on the strength of the feedback. We found that network coherence, such as the level of synchronization in population bursts, significantly suffered from virus infection. Interestingly, intervals of pathological signalling, such as asynchronous firing, alternated with stages of self-recovered “normal” population burst dynamics. Furthermore, there was direct correlation between the level of viral load and the durations of pathological signaling intervals.

## Data Availability

The datasets used and/or analysed during the current study available from the corresponding author on reasonable request.

## Code availability

Code used to produce the results presented herein is available in a public GitHub repository at https://github.com/sstasenko/
